# Evaluation of the Japanese Metabolic Syndrome Risk Score (JAMRISC): a newly developed questionnaire used as a screening tool for diagnosing metabolic syndrome and insulin resistance in Japan

**DOI:** 10.1007/s12199-016-0568-5

**Published:** 2016-10-03

**Authors:** Ce Tan, Yutaka Sasagawa, Ken-ichi Kamo, Takehiro Kukitsu, Sayaka Noda, Kazuma Ishikawa, Natsumi Yamauchi, Takashi Saikawa, Takanori Noro, Hajime Nakamura, Fumihiko Takahashi, Fumihiro Sata, Mitsuhiro Tada, Yasuo Kokai

**Affiliations:** 1 Department of General Medicine, Rumoi Municipal Hospital, 2-16-1 Sinonome, Rumoi, 077-0011 Japan; 2 Center for Medical Education, Sapporo Medical University, Sapporo, 060-8556 Japan; 3 Department of Gastroenterology, Rumoi Municipal Hospital, Rumoi, 077-0011 Japan; 4 Department of Cardiology, Rumoi Municipal Hospital, Rumoi, 077-0011 Japan; 5 NPO Rumoi Cohortopia, Rumoi, 077-0028 Japan; 6 Health Center, Chuo University, Tokyo, 162-8473 Japan; 7 Department of Neurosurgery, Rumoi Municipal Hospital, Rumoi, 077-0011 Japan; 8 Research Institute for Frontier Medicine, Sapporo Medical University, Sapporo, 060-8556 Japan

**Keywords:** JAMRISC, Logistic regression model, Questionnaire, Postprandial hyperglycemia, Insulin resistance

## Abstract

**Objectives:**

To prevent the onset of lifestyle-related diseases associated with metabolic syndrome (MetS) in Japan, research into the development of a useful screening method is strongly desired. We developed a new screening questionnaire (JAMRISC) utilizing a logistic regression model and evaluated its ability to predict the development of MetS, type 2 diabetes and other lifestyle-related diseases in Japanese populace.

**Methods:**

JAMRISC questionnaire was sent to 1,850 individuals in Rumoi, a small city in Hokkaido. We received a total of 1,054 valid responses. To maximize the target individuals accurately diagnosed with MetS, logistic regression analysis was used to generate a unique metabolic syndrome score calculation formula as taking into consideration the clinical relevance of each question item as individual coefficients.

**Results:**

The results of our comparative research utilizing both JAMRISC and Finnish Diabetes Risk Score (FINDRISC) questionnaires revealed the usefulness of JAMRISC for its ability to detect risks for MetS, pre-MetS, diabetes, and pre-diabetes. Study of disease risk detection via JAMRISC questionnaire targeting the 4283 residents of Rumoi indicated a high detection rate for pre-MetS (98.8 %), MetS (94.2 %), pre-diabetes (85.1 %) and type 2 diabetes (94.9 %). In addition, JAMRISC was useful not only as a MetS risk score test, but also as a screening tool for diagnosing insulin resistance.

**Conclusions:**

JAMRISC questionnaire is a useful instrument for the detection of early risk of not only MetS and type 2 diabetes but also insulin resistance.

## Introduction

The increased incidence of cardiovascular events accompanying the increasing number of patients with type 2 diabetes is a global issue requiring urgent measures. Retinopathy, nephropathy, and neurological disorders are well-known microvascular complications of type 2 diabetes. However, it has been recently reported that the development of macrovascular complications leading to strokes or coronary artery events starts earlier than previously believed. Namely, postprandial hyperglycemia and MetS are strongly involved in the onset of cardiovascular events [1–6].

In response to the incidence of lifestyle-related diseases associated with MetS dramatically increasing due to lifestyle changes and the rapid aging of the population in Japan, specific health checkups for MetS for Japanese residents aged 40–74 years with medical insurance were made compulsory in April 2008. However, even 7 years after the introduction of these checkups, examination rates remain much lower than the original target figures. Accordingly, it is feared that if the present situation continues, the specific health checkups will not be as effective in preventing the onset of lifestyle-related diseases and reducing medical expenses as previously projected. To make the health checkups more effective, the examination rate needs to be greatly increased. Furthermore, efficient screening methods for risk assessment need to be introduced. In this study, we first demonstrate how we developed a new health checkup questionnaire (JAMRISC). We then explain how it is more effective at detecting risk in the Japanese populace (as well as populations in other Asian countries) than FINDRISC health checkup questionnaire [7] developed in Finland 12 years ago. Finally, we describe how we used JAMRISC questionnaire when conducting a survey of early risk detection among residents aged 55–64 years in Rumoi, a small city in Hokkaido. Results of this survey indicated that JAMRISC questionnaire was useful for early disease risk detection and risk stratification.

## Subjects and methods

### Creation of JAMRISC questionnaire

**Fig. 1:**
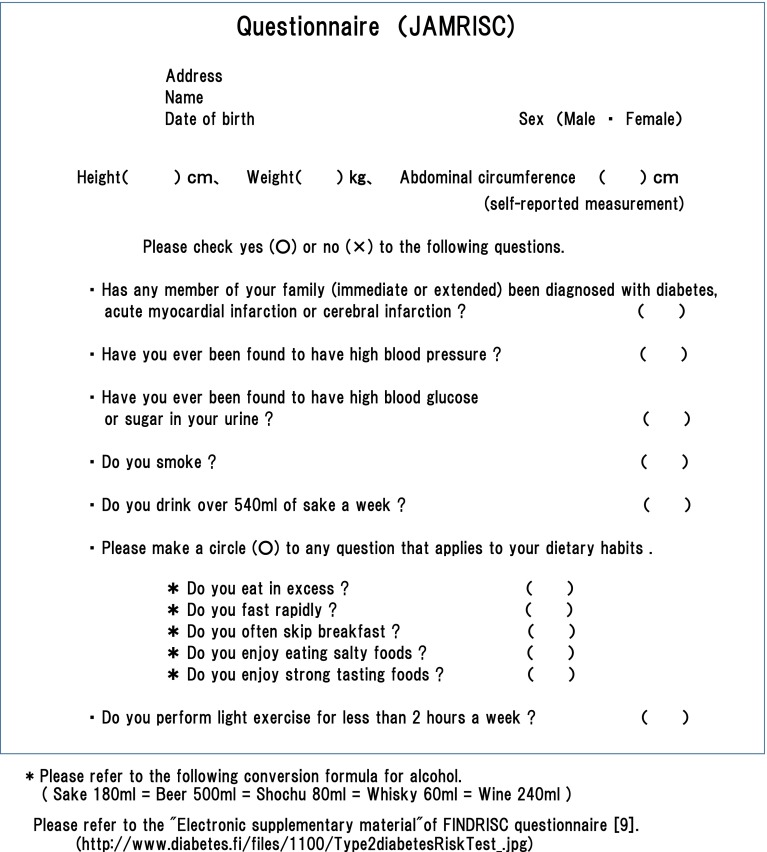
JAMRISC questionnaire

The questionnaire was composed of eleven items including age, gender, abdominal circumference (self-reported measurement around the waist), height and weight. Smoking and drinking histories were also included in addition to items related to physical activity, dietary habits, history of hypertension or hyperglycemia, and family history of myocardial infarction, stroke, diabetes (Fig. 1).

We conducted the survey from April 2007 through August 2009 with the cooperation of the residents of Rumoi City. As a result, we received a total of 1,850 responses (males 1,065; females 785). After excluding individuals undergoing treatment for a MetS-related disease and those with missing blood data items, a total of 1,054 valid responses remained. Of these 1,054 subjects, 163 males (aged 36–80 years; mean age, 57.9 years) and 30 females (aged 39–86 years; mean age, 65.0 years) were diagnosed with MetS. We adopted the Japanese MetS criteria. Individuals who suffered from central obesity (waist ≥85 cm in males, ≥90 cm in females) plus ≥2 of the following three components were defined as MetS. (1) blood pressure ≥130/85 mmHg or taking an antihypertensive, (2) fasting plasma glucose (FPG) ≥110 mg/dl, medication for diabetes, (3) serum high-density lipoprotein-cholesterol (HDL-C) <40 mg/dl, serum triglyceride ≥150 mg/dl, or medication for hyperlipidemia. Individuals who suffered from central obesity plus at least one of the conditions among these three components were defined as Pre-MetS [8].

To maximize the number of target individuals accurately diagnosed with MetS, logistic regression analysis was used to generate a unique metabolic syndrome score calculation formula taking into consideration the clinical relevance of each question item as individual coefficients. Furthermore, by multiplying the risk (probability of 0–1) predicted on the basis of this calculation formula by 100, we were able to create a total metabolic syndrome score ranging from 0 to 100 in an easy-to-understand manner.

From the receiver operating characteristic (ROC) curve, the cutoff point was set at 20 (sensitivity 0.90; specificity 0.74), with a score of lower than 20 classified as “no risk” and a score of 20 or higher classified as “at risk.” Normally, the cutoff should be set at 50; however, because of the characteristics of the health checkups, we set the cutoff at 20 to reduce false negatives and to secure results with high sensitivity and specificity.

### Comparison of the sensitivity and specificity of JAMRISC and FINDRISC questionnaires

To compare the sensitivity and specificity of JAMRISC and FINDRISC questionnaires [9], a sample of 83 subjects (aged 40–60 years), either determined to be healthy according to results of regular health checkups or definitively diagnosed with MetS, pre-MetS, type 2 diabetes, or pre-diabetes completed both the questionnaires simultaneously.

### Verification of disease risk detection in Rumoi residents through JAMRISC questionnaire

In October 2009, the questionnaire (JAMRISC) was sent via post to all 4,283 residents of Rumoi City aged 55–64 years, and responses were received from 1,915 individuals (males 855; females 1060; response rate, 44.7 %). The results indicated that 67.2 % of the subjects (males 372; females 915; total 1287) had a risk score of <20 according to the questionnaire, whereas 32.8 % of subjects (males 483; females 145; total 628) had a risk score of ≥20 indicating an “at risk” status. The 628 subjects who had a risk of ≥20 and the 218 subjects who had a risk score of <20 were recommended to undergo blood testing. The 218 subjects were extracted at random from the 1287 subjects with a risk score of <20 as a control group. As a result, a total of 846 subjects were recommended to undergo blood testing.

In accordance with the theory proposed by Matthews et al. [10], we also investigated the Homeostasis model assessment insulin resistance (HOMA-IR), an index for assessing insulin resistance calculated from FPG and fasting insulin (FIRI), and Homeostasis model assessment β cell (HOMA-β), an index that classifies insulin secretory ability. The insulin resistance index HOMA-IR was calculated using the formula FPG × FIRI ÷ 405, whereas the insulin secretory ability index HOMA-β was calculated using the formula FIRI × 360/(FPG–63).

Moreover, we adopted the diagnostic criteria of type 2 diabetes reported from the committee of the Japan Diabetes Society on the classification and diagnostic criteria of diabetes mellitus in 2010 [11]. Type 2 diabetes is diagnosed if any of the following criteria are met: (1) FPG level ≥126 mg/dl, (2) HbA1c ≥6.5 % (National Glycohemoglobin Standardization Program:NGSP). For the purpose of estimating the frequency of type 2 diabetes, “type 2 diabetes” can be substituted for the determination of “diabetic type” from a single examination. In this study, Hemoglobin A1c (HbA1c) ≥6.5% alone can be defined as “type 2 diabetes.” Generally, normal type is defined as fasting plasma glucose level of <110 mg/dl and 2-h value of <140 mg/dl in 75 g oral glucose tolerance test (OGTT). Borderline type (equal to pre-diabetes) is defined as falling between the type 2 diabetes and normal values. Subjects with borderline type correspond to the combination of impaired fasting glucose (IFG), impaired glucose tolerance (IGT) and mixed type of both IFG and IGT (IFG/IGT) noted by the World Health Organization (WHO). While IFG is diagnosed with FPG value of 110–125 mg/dl [12], IGT is diagnosed when both FPG value of <110 mg/dl and 2-h glucose levels of 140–199 mg/dl on OGTT are met [13, 14]. Mixed type of both is diagnosed when both FPG value of 110–125 mg/dl and 2-h glucose levels of 140–199 mg/dl are met.

In this study, both 75 g OGTT 2-h plasma glucose levels and casual plasma glucose level are not measured from the background of epidemiology and health screening. Although data were not shown in this study, the OGTT analysis results of 629 individuals who underwent the test at Rumoi Municipal hospital revealed that 82.1 % of individuals with FPG <110 mg/dl, HOMA-β ≥55 were equivalent to IGT. Impaired insulin action leads to postprandial hyperglycemia. Practically, impaired insulin action is hypo-secretion of insulin from the beta cell of Langerhans in the pancreas and/or decreased insulin sensitivity in peripheral tissues. Not only IGT with insulin resistance but also IFG/IGT and DM with insulin resistance were matched to the “postprandial hyperglycemia with insulin resistance”. Especially, postprandial hyperglycemia with insulin resistance was reported, which is closely related to the risk of cardiovascular diseases. So we focused on the presence of postprandial hyperglycemia with insulin resistance. Therefore, we hypothesized that individuals who met the criteria of HOMA-IR ≥1.4, FPG ≥100 mg/dl, HOMA-β ≥55 had postprandial hyperglycemia with insulin resistance. In addition, we hypothesized that IGT having insulin resistance was diagnosed when HOMA-IR ≥1.4, FPG values of 100–109 mg/dl and HOMA-β ≥55.

We then investigated the correlations between insulin resistance-related glucose metabolism disorders in which all of these criteria are met and risk scores are according to JAMRISC results.

This study was conducted with financial assistance from Rumoi City long-term care and disease risk early detection activities as part of the 2009 series of elderly health promotion activities sponsored by the Ministry of Health, Labour and Welfare. And then, all these present studies were approved by the ethics committee of the Rumoi Municipal Hospital, Rumoi, Hokkaido, Japan. Informed consent was obtained from all individual participants included in the study in written form.

## Results

### Creation of JAMRISC questionnaire

The candidates of variable which affect the occurrence for MetS are age, gender, abdominal circumference, body mass index (kg/m^2^; height and weight), smoking history, drinking history, physical activity, dietary habits, history of hypertension, history of hyperglycemia, and family history of myocardial infarction, stroke and diabetes. From these candidates, the best combination of the variables in the logistic regression model was selected using Akaike’s Information Criterion (AIC).

The set of five variables listed in Table 1 was selected as the best for explaining the risk probability against metabolic syndrome.

**Table 1:** Evaluation of the clinical relevance of the JAMRISC question items and creation of the calculation formula of the JAMRISC total risk score

Coefficients for the question items	
Gender **(**Male = l, female = 0**)**	1.3369
Abdominal circumference	0.1897
History of hypertension	1.3738
History of hyperglycemia or history of urinary sugar	
	1.5084
Exercises (yes or no) (less than 2h = 1, 2h or more = 0)	0.8768

Calculation formula of total risk score with the JAMRISC questionnaire = (1.3369 × gender ) + (0.1897 × abdominal circumference cm) + (1.3738 × history of hypertension) + (1.5084 × history of hyperglycemia / urinary sugar) + (0.8768 × exercises yes/no)

**Fig. 2:**
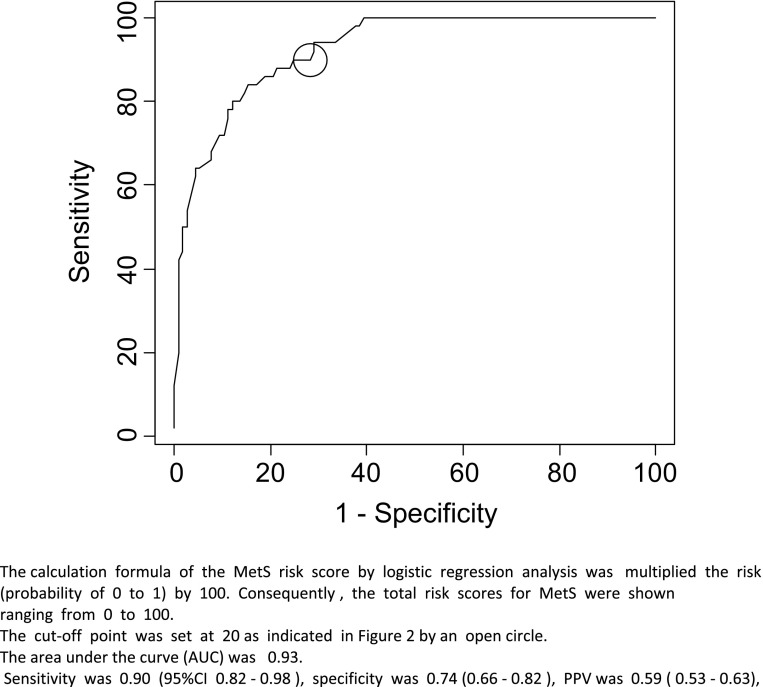
ROC curve for the detection of MetS using the JAMRISC

Coefficients in a selected optimized model were estimated to indicate the clinical relevance of each question item. When the total number of entries reached 1,054 subjects, the coefficient stabilized, and the score calculation method was considered completed. The questionnaire was composed of eleven question items, but only five explanatory items, namely gender, abdominal circumference, history of hypertension, history of hyperglycemia, and exercise habits, were required to calculate risk. The coefficients for the five items at the time of completion were as follows: gender, 1.3369 (male = 1, female = 0); abdominal circumference, 0.1897; history of hypertension, 1.3738; history of hyperglycemia, 1.5084; exercise habit, yes or no (less than 2 h = 1, 2 h or more = 0), 0.8768. Accordingly, JAMRISC total risk score was calculated by linear combination of risk factors weighted by the estimated parameters in Table 1. By translating the risk probability to percent scale, the total metabolic syndrome score ranges from 0 to 100 in an easy-to-understand manner. Next we created an ROC curve and were able to achieve a sensitivity of 90 % and specificity of 74 % when the cutoff point was set at 20, thereby completing JAMRISC (Fig. 2).

### Comparison of the sensitivity and specificity of JAMRISC and FINDRISC questionnaires

The sensitivity of JAMRISC was high, totaling 100.0 % for MetS, 90.0 % for pre-MetS, 83.3 % for type 2 diabetes, and 92.3 % for pre-diabetes. For FINDRISC, the figures were low, totaling 44.4, 0.0, 66.7, and 23.1 %, respectively. Regarding specificity, the results were somewhat low for JAMRISC, totaling 72.3 % for MetS, 63.0 % for pre-MetS, 59.7 % for type 2 diabetes, and 65.7 % for pre-diabetes, whereas the values were high for FINDRISC, totaling 100.0, 89.0, 94.8, and 92.8 %, respectively. Furthermore, an investigation of whether each questionnaire could identify individuals at risk for any of the four pathologies indicated that JAMRISC had a sensitivity of 93.1 % and specificity of 83.3 %, whereas FINDRISC had a high specificity of 100.0 % but a markedly low sensitivity of 27.6 % (Table 2).

**Table 2:** Comparison of risk detection rate between JAMRISC and FINDRISC questionnaires

Pathologies	FINDRISC		JAMRISC	
	Sensitivity	Specificity	Sensitivity	Specificity
MetS (18/83)	44.4 % (8/18)	100.0 % (65/65)	100.0 % (18/18)	72.3 % (47/65)
Pre-MetS (10/83)	0.0 % (0/10)	89.0 % (65/73)	90.0 % (9/10)	63.0 % (46/73)
Type2 diabetes (6/83)	66.7 % (4/6)	94.8 % (73/77)	83.3 % (5/6)	59.7 % (46/77)
Pre-diabetes (13/83)	23.1 % (3/13)	92.8 % (65/70)	92.3 % (12/13)	65.7 % (46/64)
Overall risk for above four pathologies^a^ (29/83)	27.6 % (8/29)	100.0 % (54/54)	93.1 % (27/29)	83.3 % (45/54)

A sample of 83 subjects (aged 40–60 years) definitively diagnosed as healthy or with MetS, pre-MetS, type 2 diabetes, or pre-diabetes according to the results of regular health checkups completed both JAMRISC and FINDRISC questionnaires simultaneously

^a^JAMRISC could detect individuals with any risks related to type 2 diabetes and MetS with a sensitivity of 93.1 % and a specificity of 83.3 %, whereas FINDRISC offered high specificity (100.0 %), but markedly low sensitivity (27.6 %)

### Verification of disease risk detection via JAMRISC targeting the residents of Rumoi

We sent questionnaires to 4,283 residents of Rumoi City aged 55–64 years (males 2,008; females 2,275) whose data were extracted from the basic resident register. Valid responses were received from 855 males (42.6 %) and 1,060 females (46.6 %) with a total response rate of 44.7 %.

No significant difference was observed between males and females concerning the number of questionnaires sent or responses received. We calculated the risk for the 1,915 subjects from whom responses were received and found that 1,287 subjects (67.2 %) had a risk score of <20, indicating “no risk,” whereas 628 subjects (32.8 %) had a risk score of 20 or higher, indicating that they were “at risk”. Among the 1,915 subjects, 217 (11.3 %), 241 (12.6 %), and 170 subjects (8.9 %) had scores of 20–49, 50–89, and 90–100, respectively. The 628 subjects who had a risk score of ≥20 and the 218 subjects who had a risk score of <20 were recommended to undergo blood testing. As a result, the 298 subjects who had a risk score of ≥20 and the 98 subjects who had a risk score of ≥20 underwent blood testing (Table 3).

**Table 3:**
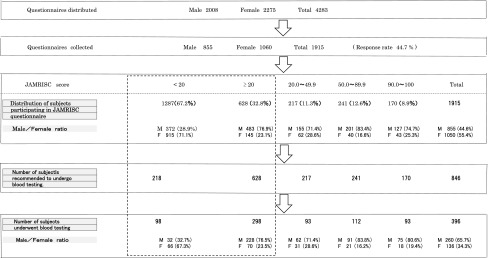
Timetable of JAMRISC questionnaire utilized to direct disease development risk in Rumoi residents aged 55–64 years

As shown in Table 4, study of disease risk detection via JAMRISC questionnaire indicated a high detection rate for pre-MetS (98.8 %), MetS (94.2 %), pre-diabetes (85.1 %) and type 2 diabetes (94.9 %). Furthermore, the results of blood testing revealed that the mean HOMA-IR was 1.15 for subjects with a questionnaire score less than 20 (males, 32.7 %), 1.67 for subjects with a score of 20–49 (males, 71.4 %), 1.66 for subjects with a score of 50–89 (males 83.8 %), and 2.25 for subjects with a score of 90–100 (males 80.6 %), indicating strong insulin resistance. Accordingly, insulin resistance tended to increase as the risk score increased. Therefore, insulin resistance intensity was set at three levels: HOMA-IR ≥1.4, HOMA-IR ≥2.0 and HOMA-IR ≥3.0, and insulin resistance detection rates were investigated for each risk score. The results indicated that 87.1 % of subjects with a risk score of ≥20 were HOMA-R ≥1.4, 91.2 % were HOMA-IR ≥2.0, and 92.3 % were HOMA-IR ≥3.0. Accordingly, this demonstrated that the JAMRISC risk evaluation could be used to determine insulin resistance with the cutoff point set at 20 and that even slight resistance as denoted by HOMA-IR ≥1.4 could be detected (Table 5).

**Table 4:**
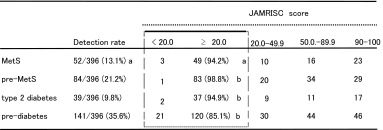
Validation of the risk detection rate by the JAMRISC questionnaire for MetS, pre-MetS, type 2 diabetes, and pre-diabetes in 396 subjects that underwent blood testing

^a^Among the 396 subjects who underwent blood testing, 52 subjects (equivalent to 13.1 %) were diagnosed with MetS, among whom 49 (94.2 %) exhibited the risk scores of ≥20

^b^High detection rates were also shown for pre-MetS, type 2 diabetes and pre-diabetes

**Table 5:**
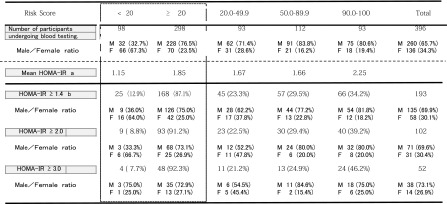
Correlation between the risk score calculated with JAMRISC and the degree of insulin resistance

^a^The results of blood testing revealed that the mean HOMA-IR was 1.15 for subjects with a questionnaire score of <20 (males, 32.7 %), 1.67 for subjects with a score of 20–49 (males, 71.4 %), 1.66 for subjects with a score of 50–89 (males, 83.8 %), and 2.25 for subjects with a score of 90–100 (males, 80.6 %), indicating strong insulin resistance. Accordingly, insulin resistance tended to increase as the risk score increased

^b^Insulin resistance intensity was set at three levels: HOMA-IR ≥1.4, HOMA-IR ≥2.0, HOMA-IR ≥3.0, and insulin resistance detection rates were investigated for each risk score. The results indicated that 87.1% of subjects with a risk score of ≥20 were HOMA-R ≧1.4, 91.2 % were HOMA-IR ≧2.0, and 92.3 % were HOMA-IR ≧3.0

As shown in Table 6, the rate of subjects with “postprandial hyperglycemia with insulin resistance” which included IGT, IFG/IGT and type 2 diabetes increased with increasing risk scores.

**Table 6:**
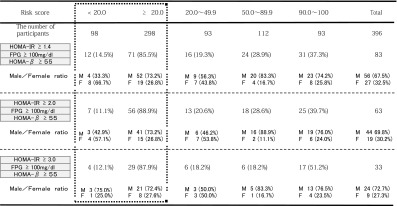
Correlation between the rates of subjects with postprandial hyperglycemia with insulin resistance and the risk score calculated with JAMRISC

Although data were not shown in this study, the OGTT analysis results of 629 individuals who underwent the test at Rumoi Municipal hospital revealed that 82.1% of individuals with FPG <110mg/dl, HOMA-β ≥55 were equivalent to IGT

In addition, recent epidemiological data in Japan show that subjects with FPG values of 100 to 109mg/dl, which are in the normal range, develop type 2 diabetes at a higher rate than subjects with FPG values <100mg/dl

Moreover, FPG values of 100 mg/dl are seem to be corresponding to 2-hr values of 140 mg/dl in 75 g OGTT approximately (J. Japan Diab. Soc. 51(3): 281-283, 2008). With those reports as a background, we hypothesized that IGT having insulin resistance was diagnosed when HOMA-IR ≥ 1.4, FPG values of 100-109mg/dl and HOMA-β ≥ 55

For the purpose of target all subjects exhibited “postprandial hyperglycemia with insulin resistance” within IGT, IFG/IGT and type 2 diabetes, we decided to describe FPG values of ≥100 mg/dl

Therefore, we hypothesized that individuals who met the criteria of HOMA-IR ≥ 1.4, FPG ≥100 mg/dl, HOMA-β ≥ 55 had postprandial hyperglycemia with insulin resistance

## Discussion

From 2003 to 2025, it is projected that there will be a 72 % increase in type 2 diabetes worldwide [15, 16]. It is also predicted that the incidence of MetS in addition to type 2 diabetes will rapidly increase in Japan and other Asian countries (Korea, China, and India), as well as in developing countries.

Approximately 12 years ago, a simple questionnaire called FINDRISC that was scored on the basis of the Framingham Study was developed in Finland in Northern Europe. In the initial study, it was reported that development of type 2 diabetes was suppressed in the intervention group by 58 % compared with the non-intervention group [17]. The questionnaire was developed to screen individuals who had a high risk of developing type 2 diabetes in the future and reduce its onset of incidence through early intervention [18]. These results were later confirmed in various countries and the questionnaire is now accepted and utilized worldwide [19, 20]. FINDRISC was first developed as a diabetes risk test. Moreover, it has recently come to be used to assess MetS risk [21, 22]. Accordingly, this questionnaire could greatly increase the rate at which people undergo health examinations due to its simplicity, low cost, and non-invasiveness. However, because the dietary habits and physique of Japanese people are greatly different from those of Western people, FINDRISC might not necessarily be as effective when applied to Japanese people. The results of our comparative research, which indicated that FINDRISC risk detection rate was markedly low, suggest that FINDRISC should be modified to suit Japanese people and that MetS risk questionnaires should be developed specifically for the Japanese.

In developing countries in Asia, the incidence of cardiovascular events is expected to rise dramatically with the rapid increase in lifestyle-related diseases associated with MetS and diabetes. In this study, therefore, we developed a health checkup questionnaire for Japanese people (JAMRISC) that used a different method from that of FINDRISC that was able to detect not only type 2 diabetes and MetS, but also pre-diabetes and pre-Mets conditions with high accuracy.

Screening with currently available questionnaires, including FINDRISC, usually involves evaluation with whole numbers indicating the clinical relevance for each question item (e.g., 0 point, 1 point, 2 points, and so forth), and the scores for each item are then totaled to create an overall risk score. We, however, adopted a method different from the conventional ones to calculate MetS risk.

First, we conducted a questionnaire survey for a population in which individuals with MetS (meeting Japanese criteria) had already been clarified. Next, without revealing subjects already identified as having MetS, logistic regression analysis was used to estimate clinical relevance for each question item to achieve the highest accuracy possible. After that, the number of participants in the population was gradually increased and, once the number of subjects reached 1,054, the risk calculation formula was completed at the point when question item coefficient fluctuation decreased and stabilization was achieved.

The JAMRISC questionnaire had eleven question items. Five of these were explanatory items, and the remaining six were considered to have been explained by these five items. It should be noted that abdominal circumference was not allocated to possible responses such as 85 cm or approximately 90 cm, but reflected an actual measurement of abdominal circumference in centimeters and was used to demonstrate risk transition with continuity.

In general, although screening via questionnaires is simple, easy to participate in, and can be done at home because it does not require blood testing, there is a significant disadvantage that forced its low risk detection. In contrast, the JAMRISC questionnaire offered high detection with a sensitivity of 94.2 % for MetS in this study.

We confirmed that JAMRISC had had a higher sensitivity than and comparable specificity to FINDRISC. In addition, we also demonstrated that the JAMRISC questionnaire could also detect insulin resistance, which occurs at an even earlier stage in disease progression. The ability to detect not only pre-diabetes and pre-MetS but also mild insulin resistance may lead to the prevention of type 2 diabetes, MetS, as well as severe lifestyle-related diseases such as cardiovascular disease [23, 24], Alzheimer-type dementia [25, 26], and cancer [27, 28].

Recently, many reports have indicated that insulin resistance itself is closely related to cardiovascular events [29–33]. A GAMI study conducted by Ryden et al. [34, 35] found that one-third of patients hospitalized for acute myocardial infarction were diagnosed with type 2 diabetes, one-third had postprandial hyperglycemia diagnosed with IGT or IFG/IGT, and the remaining third had normal glucose metabolism. Some of these patients with normal glucose metabolism may have been in a high-risk group exhibiting very mild insulin resistance [36, 37]. Therefore, we decided to use HOMA-R ≥1.4 as an indicator of “the presence of insulin resistance” so as to determine the appearance of even slight insulin resistance and thus prevent exacerbation.

The spread of simple and low-cost methods of screening with high risk detection sensitivity such as JAMRISC could contribute to the prevention of the onset of lifestyle-related diseases associated with MetS and type 2 diabetes. JAMRISC was found to not only exhibit high precision for detecting the presence or absence of risk, but also offered possibilities for stratifying low to high risk levels, therefore, suggesting that it could be an extremely useful method for screening the risk of lifestyle-related diseases.
